# Editorial: Molecular basis of stress resistant signal transduction in plants: a biotechnological intervention to develop climate-resilient crops

**DOI:** 10.3389/fpls.2024.1356520

**Published:** 2024-03-01

**Authors:** Zhaolong Xu, Girdhar Kumar Pandey, Zulfiqar Ali, Dayong Zhang

**Affiliations:** ^1^ Provincial Key Laboratory of Agrobiology, The Jiangsu Provincial Infrastructure for Conservation and Utilization of Agricultural Germplasm, Jiangsu Academy of Agricultural Sciences, Nanjing, China; ^2^ Department of Plant Molecular Biology, University of Delhi, New Delhi, India; ^3^ Department of Plant Breeding and Genetics, Faculty of Agriculture, University of Agriculture, Faisalabad, Pakistan; ^4^ State Key Laboratory of Crop Genetics & Germplasm Enhancement and Utilization, Nanjing Agricultural University, Nanjing, China

**Keywords:** abiotic stress, signal transduction, ion homeostasis, climate change, stress resistant crops

Plants live in constantly changing environments, which often have various impacts and pressures on plant growth. Due to their fixed nature, various pressures on plants often affect their growth and development to varying degrees. These adverse environmental conditions include biological stress, such as pathogen infections and attacks from herbivores, as well as physical, chemical, and other abiotic stresses, such as drought, waterlogging, insufficient or excessive light, as well as nutrient deficiency or excess, and excessive salt or toxic metals in the soil. Drought, salinity, and temperature stress are the main environmental factors that affect the geographical distribution of plants in nature, limit agricultural plant productivity, and threaten food security. Climate change exacerbates the adverse effects of these abiotic stresses, and it is predicted that climate change will lead to an increase in the frequency of extreme weather events (Fedoroff et al., 2010). How plants perceive stress signals and adapt to adverse environments is a fundamental biological issue.

Previous studies have shown that after initial exposure or perception of these stressors, many signaling pathways are activated to transduce stress signals and prepare plants to cope with stress conditions. The signals generated in these pathways include various plant hormones such as abscisic acid (ABA), second messengers such as calcium ions (Ca^2+^), and a large number of signaling proteins such as receptors, kinases, phosphatases, transcription factors, channels, and transporters, thereby enhancing the plant’s tolerance to stress (Zeng et al., 2015). This is crucial for agricultural productivity and environmental sustainability, as crops with poor stress resistance consume too much water and fertilizer, resulting in a huge burden on the environment. It is helpful to distinguish between primary and secondary stress signals caused by insufficient water or excessive salt for drought and salt stress. The main signal caused by drought is high osmotic stress, commonly referred to as osmotic stress, because low osmotic conditions are usually not a significant problem for plant cells. Salt stress has both osmotic and ion or ion toxicity effects on cells (Leung et al., 2023). The secondary effects of drought and salt stress are complex, including oxidative stress; Damage to cellular components such as membrane lipids, proteins, and nucleic acids; and metabolic dysfunction. Although some cellular responses are caused by primary stress signals, others are mainly caused by secondary signals. Therefore, drought and salt have unique and overlapping signals. An important characteristic of drought and salt stress is that high osmotic signals lead to the accumulation of plant hormone abscisic acid (ABA), resulting in many adaptive responses in plants (Zhou et al., 2014).

The various stress responses and molecular mechanisms mentioned above have been extensively studied in the model plant Arabidopsis to understand the roles of some of these components in stress perception, signal transduction, and response generation for adaptation or tolerance to specific stresses. However, the functions of many stress signal components have not been fully determined, especially the mechanisms of stress signal transduction in crops need further exploration. In addition, it is necessary to utilize existing knowledge to develop crops that can adapt to climate change. With advanced genetic and genomic engineering tools, it is possible to alter stress signaling pathways, thereby developing crop varieties that can grow and reproduce in stress environments without losing crop productivity and yield. Therefore, understanding the detailed molecular mechanisms of crop stress signals has enormous scope and potential.

Based on the summary of this topic, we can say that although the vast majority of plants cannot move, they basically have a special set of strategies to resist abiotic stress, as shown in **Figure 1**. When there is external stress, the calcium ions in the plant roots or leaves act as second messengers to quickly transmit stress information, initiate ion and osmotic pressure regulation schemes, and stabilize ions, supply energy Relieve internal pressure in cells by eliminating reactive oxygen species. Simultaneously, through cascade reactions, regulating the opening and closing of stomata, reducing water loss in stress responses, etc.

**Figure 1:**
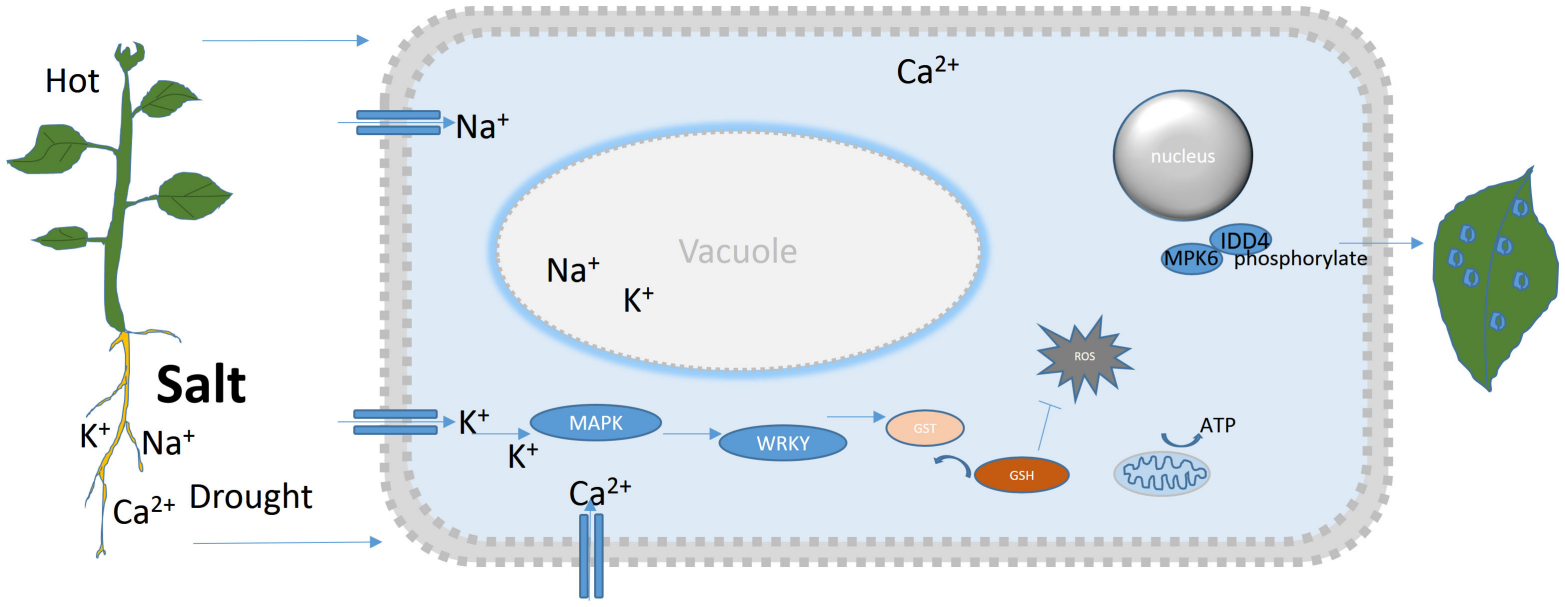
Strategies for plants to resist abiotic stresses. When plants are subjected to salt stress or other abiotic stresses, their strategy is to absorb more substances with less damage, such as potassium ions, in order to reduce the absorption of harmful substances such as sodium ions, and activate various protective mechanisms, such as the clearance of reactive oxygen species, the number and opening of stomata, etc.

This Research Topic aims to gather scientific findings on plant stress signals and adaptation from leading plant biologists worldwide. This includes identifying the response molecules of different plants under various pressures, analyzing the function of basic signal transduction proteins, and studying various products that play a crucial role in stabilizing cell function under stress environments. In order to better respond to global food supply shortages, we particularly welcome research on basic crops, including rice, wheat, corn, soybeans, barley, and other food crops. We hope that this Research Topic can provide a communication platform for most plant stress resistance researchers, and provide specific overall basis for crop stress resistance research and screening of new stress resistant varieties in the near future.

In this Research Topic, an original study on why the dryland rice variety 17SM-19 has high salt tolerance had been published. Through comprehensive analysis of transcriptome and metabolome, the response of rice seedlings to salt stress was determined (Zhou et al.). The results indicate that the salt tolerance of the seedlings of the local variety 17SM-19 of upland rice involves many molecular mechanisms, such as osmotic regulation, ion balance, and reactive oxygen species clearance. The mechanism of high salt stress resistance in salt tolerant varieties was analyzed from the perspective of gene expression, which not only provides germplasm resources for cultivating salt tolerant rice, but also provides a theoretical basis for gene aggregation breeding. Similar studies have also been conducted on chickpeas, comparing transcriptome, proteome, and metabolome analyses of two chickpeas genotypes that respond significantly to drought stress, in order to gain a deeper understanding of the molecular mechanisms underlying drought stress response/tolerance. The differences in glycolysis/gluconeogenesis, galactose metabolism has been analyzed, starch and sucrose metabolism among different tolerance types, and revealed that co expressed genes, proteins, and metabolites involved in phosphatidylinositol signaling, glutathione metabolism, and glycolysis/gluconeogenesis pathways were more prominent in stress tolerant types (Singh et al.).

Barley, as an important grain and economic crop, not only affects people’s quality of life, but also serves as a pioneer plant for improving saline alkali land. A study in this Research Topic shows that barley has strong salt tolerance, however, its genetic basis is not fully understood, especially during the seedling stage (Xu et al.). Studied the ion changes in barley germplasm under control and salt stress. The genome-wide association study (GWAS) analysis revealed 54 significant SNPs on 7 chromosomes. These SNPs are related to ion homeostasis traits, sodium (Na^+^) and potassium (K^+^) content, and Na^+^/K^+^ ratio, and may be hot spots for exploring and identifying salt tolerant candidate genes. In addition, 616 unique candidate genes were screened around significant SNPs, which are associated with transporters, protein kinases, binding proteins, and other unknown functional proteins. These results provide candidate genes responsible for salt response in barley and offer new ideas for studying this genetic basis in similar crops.

Two other studies on cotton salt resistance identified important genes related to salt tolerance using different methods and plant materials (Sun et al.). The former had 24 genes and the latter had 120 genes (Ju et al.) both of which were not extensively studied. The author also infers that these genes play a crucial role in the response of upland cotton to salt stress, helping to cultivate salt tolerant cotton varieties that can grow on saline alkali soil; This helps to provide new insights into the molecular mechanisms underlying potassium enhanced salt adaptability in cotton, laying a theoretical foundation for the improvement and innovation of high-quality cotton germplasm.

In summary, abiotic stress is the main limiting factor that poses a serious threat to agricultural production. Traditional breeding has significantly improved crop productivity, but due to the multi gene nature of abiotic stress, traditional breeding has reached its maximum capacity. Biotechnological methods can provide new opportunities for the production of crops that can adapt to rapidly changing environments and still produce high yields under severe environmental pressure conditions. Many genes related to stress have been identified and manipulated to produce stress tolerant plants, which may lead to further increases in food production in most countries around the world.

## Author contributions

ZX: Conceptualization, Data curation, Formal analysis, Funding acquisition, Methodology, Project administration, Software, Supervision, Validation, Visualization, Writing – original draft, Writing – review & editing. GP: Writing – original draft. ZA: Writing – original draft. DZ: Writing – original draft.
